# Osteocalcin Is Not Associated with the Risk of Type 2 Diabetes: Findings from the EPIC-NL Study

**DOI:** 10.1371/journal.pone.0138693

**Published:** 2015-09-29

**Authors:** Sabine R. Zwakenberg, Caren M. Gundberg, Annemieke M. W. Spijkerman, Daphne L. van der A, Yvonne T. van der Schouw, Joline W. J. Beulens

**Affiliations:** 1 Julius Center for Health Sciences and Primary Care, University Medical Center Utrecht, Utrecht, the Netherlands; 2 Department of Orthopaedics, Yale University School of Medicine, New Haven, Connecticut, United States of America; 3 Center for Nutrition, Prevention and Health Services, National Institute for Public Health and the Environment, Bilthoven, the Netherlands; Baylor College of Medicine, UNITED STATES

## Abstract

**Objective:**

To investigate whether total osteocalcin (tOC), uncarboxylated osteocalcin (ucOC) and percentage of uncarboxylated osteocalcin (%ucOC) are associated with the risk of type 2 diabetes.

**Methods:**

This nested case control study included 1,635 participants, 833 incident diabetes cases and 802 non-diabetic control participants, aged 21–70 years from the EPIC-NL cohort. Baseline concentrations of tOC, ucOC and %ucOC were assessed. During 10 years of follow-up, diabetes cases were self-reported and verified against information from general practitioners or pharmacists. The association between the different forms of osteocalcin and diabetes risk was assessed with logistic regression adjusted for diabetes risk factors (waist circumference, age, sex, cohort, smoking status, family history of diabetes, hypertension, alcohol intake, physical activity and education) and dietary factors (total energy intake and energy adjusted intake of fat, fiber, protein and calcium).

**Results:**

TOC concentration was not associated with diabetes risk, with an odds ratio (OR) of 0.97 (0.91–1.03) for each ng/ml increment after adjustment for diabetes risk factors and dietary factors. No association between ucOC and %ucOC and the risk of diabetes was observed either. In sex stratified analyses (P interaction = 0.07), higher %ucOC tended to be associated with an increased risk of type 2 diabetes in a multivariable model in women (OR 1.05 for each increment of 5% ucOC (1.00–1.11), P_trend_ = 0.08), but not in men (OR 0.96 for each increment of 5% ucOC (0.88–1.04)). When waist circumference was replaced by body mass index, none of the osteocalcin forms were associated with the risk of type 2 diabetes in the final model among both women and men.

**Conclusions:**

Available evidence suggests that tOC, ucOC and %ucOC are each not associated with the risk of type 2 diabetes. However, more large-scale cohort studies are needed to clarify the presence of any association between the different forms of osteocalcin and the risk of type 2 diabetes.

## Introduction

Osteocalcin is a vitamin K dependent protein, which is primarily known for its role in bone mineralization. Vitamin K is an essential co-factor for carboxylation of osteocalcin and carboxylation of total osteocalcin (tOC) increases the proportion of carboxylated osteocalcin (cOC) and lowers the proportion of uncarboxylated osteocalcin (ucOC) [1]. Due to this essential role, percentage uncarboxylated osteocalcin (%ucOC) is often used as marker for vitamin K status with high %ucOC indicating a low vitamin K status [2,3].

In recent years, an accumulating body of evidence has suggested that osteocalcin plays a role in the regulation of glucose metabolism [4]. However, different studies ascribe this effect to different forms of osteocalcin. Mouse studies indicate that ucOC is the active form in this respect but studies in human are conflicting [5–9].

To date, different cross-sectional studies have investigated the association between tOC and diabetes risk, showing a pooled risk estimate of 0.23 (0.12–0.46) for the highest quartile compared to the lowest quartile [10]. However, only three prospective cohort studies were performed on this relation showing inconsistent results regarding the role of tOC [11–13]. The association between ucOC levels and the risk of type 2 diabetes also remains inconclusive [4,12].

Studies on the percentage ucOC are scarce. Only one small scale prospective study investigated the association between the ratio of ucOC with tOC and diabetes incidence in men (n = 126), but this study lacked adequate power to detect significant associations [12]. With the exception of this small scale study, all studies measured the absolute level of different osteocalcin forms. Because the differing assays that measure the various forms are not standardized equivalently, they cannot be used to estimate %ucOC [5]. The hydroxyl apatite (HAP) binding method first separates the carboxylated and uncarboxylated forms of osteocalcin. A subsequent competitive assay that recognized both cOC and ucOC, equivalently allows the calculation of %ucOC. Only one cross-sectional study used the HAP assay to investigate the association between osteocalcin and diabetes and showed that a higher %ucOC was associated with a higher risk of type 2 diabetes [14].

To date, no prospective studies have evaluated the association between %ucOC and incidence type 2 diabetes, using an adequate measurement to determine %ucOC. Furthermore, a recent published meta-analysis of prospective studies showed a null association between tOC and type 2 diabetes [10]. They also stated that large-scale prospective studies are needed to detect whether osteocalcin may be useful in the prevention of diabetes.

Therefore, this study aims to prospectively investigate the association between different forms of osteocalcin, tOC, ucOC and %ucOC as assessed by the HAP assay, and the risk of type 2 diabetes. We will also investigate whether %ucOC is an adequate marker of vitamin K intake.

## Materials and Methods

### Study population

This case-control study was nested within the EPIC-NL cohort, a prospective cohort of the two Dutch contributions (Prospect-EPIC and MORGEN-EPIC) to the European Prospective Investigation into Cancer and Nutrition (EPIC). The cohorts were set up simultaneously in 1993–1997. The Prospect-EPIC study includes 17,357 women between the ages 49 to 70 who were living in Utrecht and vicinity. The MORGEN-EPIC cohort consists of 22,654 adults aged 21–64 years who were selected from random samples of three Dutch towns; Amsterdam, Maastricht and Doetinchem. All participants provided written informed consent before study inclusion. The study complies with the Declaration of Helsinki and was approved by the Institutional Review Board of the University Medical Center Utrecht and the Medical Ethical Committee of TNO Nutrition and Food Research (MORGEN-EPIC). The design and rationale of EPIC-NL are described elsewhere [15].

During 10 years of follow-up, 918 incident type 2 diabetes cases were documented. The control group (n = 900) consisted of participants who were free from diabetes at the end of follow up. The control group was randomly drawn from the remainder of the baseline cohort excluding incident cases of type 2 diabetes. For this study, an equal amount of participants was drawn from the Prospect-EPIC as from the MORGEN-EPIC cohort. None of the participants were taking vitamin K antagonist medication. After exclusion of participants with missing osteocalcin levels (n = 183), a total number of 1,635 participants remained for analysis (833 incident diabetes type 2 cases and 802 controls).

### Osteocalcin

All participants donated a 30 ml non-fasting blood sample at baseline. TOC and ucOC were measured in serum samples with the hydroxyl apatite (HAP) binding assay. This HAP method is based on lower affinity of ucOC for hydroxyapatite compared to the cOC [5]. The method has been used extensively and provides discrimination between the carboxylated and uncarboxylated form. The total amount of osteocalcin was presented as tOC in ng/ml, ucOC in ng/ml and %ucOC. Detailed information of this measurement is described in the S1 File.

### Type 2 diabetes

Three different sources were used to ascertain potential diabetes cases: self-report, linkage with hospital discharge registries and a self-administered urine dipstick test. The diagnosis diabetes was self-reported in follow-up questionnaires every three to five years. Participants were asked whether or not diabetes was diagnosed, in what year, by whom and which treatment they received. Secondly, among Prospect participants only, a urinary glucose strip test was sent out with the first follow-up questionnaire for detecting potential diabetes cases with glucosuria. Finally, diabetes was diagnosed based on linkage with the database of the Dutch Center for Health Care Information, which holds a standardized computerized register of hospital discharge diagnoses [16]. Potential diabetes cases detected with one of these methods were verified with questionnaires mailed to the general practitioner or pharmacist of the participant. For 89% of the potential diabetes cases, verification information was available. Finally, 72% were verified as having type 2 diabetes [16].

### Other measurements

At baseline, participants completed general questionnaires about demographic and lifestyle characteristics and risk factors for chronic diseases. Smoking status was categorized as current, past or never smoker. Family history of diabetes was defined as parents without diabetes or at least one parent with diabetes. Education was classified as low, middle and high education. Physical activity was measured with the validated Cambridge Physical Activity Index, and was categorized as inactive, moderately inactive, moderately active and active [17]. Hypertension was defined as systolic blood pressure above 140mmHg or diastolic blood pressure above 90 mmHg [15].

Dietary intake was assessed with a semi-quantitative validated food frequency questionnaire (FFQ). The FFQ estimates the average daily consumption of 178 foods during the year preceding enrolment. The Dutch food composition table (1996) was used to calculate nutrient intake. However, this table does not include information on the vitamin K content of foods. Therefore, the concentrations of phylloquinone and menaquinones in common Dutch foods were measured at the Biochemistry Laboratory, Maastricht University [18]. For other foods, published data were used to update the vitamin K database [18–20]. In total, vitamin K contents of 260 foods were collected.

The FFQ has been validated against 12 monthly 24-hour dietary recalls in 121 individuals. The validity of phylloquinone against these 24-hour recalls was relatively low (r = 0.26 and 0.19 in men and women respectively). The validity of menaquinones intake was reasonable with correlations coefficients of 0.60 and 0.48 in men and women. The intake of all nutrients was adjusted for total energy intake with the regression residual method [21].

### Statistical analyses

Baseline categorical data were presented as percentages and continuous data as means and standard deviation (SD) except for skewed variables, which were presented as medians and interquartile ranges. Since data were missing at random, missing values were multiple imputed with 5 imputations and combined according to Rubin’s rules [22]. The imputation model included diabetes status, osteocalcin levels and all possible confounders as predictors of missing values.

We used logistic regression analysis to estimate odds ratio (ORs) of type 2 diabetes for quartiles of tOC, ucOC and %ucOC. Exposure variables were also modeled continuously to calculate the OR for type 2 diabetes by one ng/ml change in tOC and ucOC. For ease of interpretation, %ucOC is modelled per 5% increment of %ucOC. The selection for potential confounders was based on literature, and defined as confounder when it changed the association with more than 10%. We adjusted for type 2 diabetes risk factors and dietary factors in three separate models. The first model was adjusted for cohort, sex, age and waist circumference. In the second model we additionally adjusted for other diabetes risk factors: smoking status, physical activity, hypertension (yes/no), alcohol intake measured with the FFQ, family history of diabetes (yes/no) and level of education. In the third model, additional adjustments were made for dietary factors: total energy intake and energy adjusted intakes of saturated fat, mono- and polyunsaturated fat, fiber, protein and calcium.

P-values for linear trends were estimated by including the median value of each quartile as a continuous variable in the model. To explore the presence of non-linear associations, a quadratic term (the square of the median in the model) was added together with the linear term. In certain cases (model 1 of tOC and all models of %ucOC) this term was significant and the shape of the association was further explored using spline regression analyses. Since spline regression analyses did not confirm a significant non-linear association (p = 0.18) for any of the variables, we present the results for the quartiles and continuous analyses here. Effect modification by sex was checked for all forms of osteocalcin, by including interaction terms in the model. In case of a significant interaction (p<0.05), the analyses were stratified for this variable. Sensitivity analyses were performed to assess whether the associations changed when waist circumference was replaced by body mass index (BMI), because BMI is an important determinant of bone remodeling [23]. Finally, the association between dietary intake of vitamin K, measured with the FFQ, and %ucOC was assessed by Spearman correlation coefficients, to assess whether osteocalcin is a reliable marker for vitamin K intake. Furthermore we investigated the associations between the different forms of osteocalcin with Spearmann correlations (r_s_).

The software package SPSS (version 20 for Windows) and SAS 9.2 were used for statistical analyses and a two tailed p value of <0.05 was considered to be statistically significant.

## Results

Baseline characteristics of diabetes cases and controls are shown in Table 1. Participants with diabetes had less favorable metabolic profiles compared to participants without diabetes. At baseline, those who developed diabetes during follow up had a higher BMI, waist circumference and more often had hypertension. Diabetes cases were less active compared with the controls and alcohol intake was higher in the control group compared to the diabetes cases.

**Table 1:** Baseline characteristics according to diabetes incidence after 10 year follow up of 1,635 Dutch adults participating in the EPIC-NL study.

Characteristics	Diabetes	Non-diabetes
	n = 833	n = 802
Men (%)	20.8	24.9 *
Age (years)	57.0 (51.8–62.3)	50.5 (41.3–57.1) *
BMI (kg/m^2^)	29.7 ± 4.5	25.3 ± 3.8 *
Waist circumference (cm)	96.6 ± 11.4	84.4 ± 11.5 *
Physically inactive (%/n)	14.3	8.4 *
Current smoker (%)	26.2	31.8
Alcohol intake (g/day)	1.5 (0.1–10.5)	5.8 (0.7–16.9) *
High education (%)	8.9	21.3 *
Family history of diabetes (%)	38.4	18.0 *
Postmenopausal (%/women)	58.2	36.3
Systolic BP (mmHg)	140.0 ± 21.9	124.7 ± 19.0 *
Diastolic BP (mmHg)	83.6 ± 10.5	71.0 ± 10.5 *
Hypertension (%)	68.3	34.3 *
Cholesterol (ng/ml)	5.9 ± 1.1	5.5 ± 1.0 *
TOC (ng/ml)	4.0 (2.9–5.5)	4.4 (3.2–5.7) *
UcOC (ng/ml)	1.8 (1.2–2.7)	2.0 (1.3–2.9) *
%ucOC	47.00 ± 15.8	46.92 ± 14.3
**Diet**		
Energy (kcal/day)	1815 (1533–2219)	1930 (1610–2375) *
Vitamin K (μg/day)	227.0 (167–300)	204.2 (156–574) *
Phylloquinone (μg/day)	194.3 (136–266)	173.9 (126–243) *
Menaquinones (μg/day)	30.0 (22–39)	27.7 (20–37) *
Vitamin C (mg/day)	110.6 ± 45	108.7 ± 46 *
Calcium (mg/day)	1120.6 ± 397	1058.4 ± 356 *
Fiber (g/day)	23.8 ± 5	23.4 ± 5
Protein (g/day)	79.4 ± 12	75.4 ± 11 *

* Significant different compared to diabetes patients, p<0.05

Data are given as mean ± SD or median (interquartile range) or percentages.

All nutrients are measured with the FFQ and all values are energy adjusted except energy intake.

Table 2 shows the association between different measures of osteocalcin (tOC, ucOC and %ucOC) and the risk of type 2 diabetes. TOC was not associated with the risk of type 2 diabetes in the three different models with an OR_Q4_ of 0.97 (0.69–1.36) in the first model and an OR_Q4_ of 1.02 (0.71–1.48) in the third model. Absolute levels of ucOC were not associated with risk of type 2 diabetes either (model 3, OR_Q4_ 0.88 (0.61–1.27)). Furthermore, %ucOC was not associated with the risk of type 2 diabetes (model 3, OR_Q4_ 1.09 (0.76–1.57)).

**Table 2:** Total osteocalcin, uncarboxylated osteocalcin and percentage uncarboxylated osteocalcin and the risk of type 2 diabetes among 1,635 Dutch men and women.

**Total osteocalcin**						
	**Per one ng/ml**	**Q1**	**Q2**	**Q3**	**Q4**	**P**_**trend**_
TOC (ng/ml)		2.3 ± 0.5	3.7 ± 0.6	4.9 ± 0.4	7.5 ± 2.1	
Model 1	0.97 (0.92–1.02)	1.0	1.21 (0.87–1.69)	0.58 (0.49–0.69)	0.97 (0.69–1.36)	0.29
Model 2	0.96 (0.90–1.02)	1.0	1.25 (0.88–1.79)	0.60 (0.42–0.85)	0.95 (0.66–1.36)	0.24
Model 3	0.97 (0.91–1.03)	1.0	1.34 (0.93–1.93)	0.60 (0.42–0.86)	1.02 (0.71–1.48)	0.39
**Uncarboxylated osteocalcin**						
	**Per one ng/ml**	**Q1**	**Q2**	**Q3**	**Q4**	**P**_**trend**_
ucOC (ng/ml)		0.9 ± 0.4	1.6 ± 0.2	2.3 ± 0.2	3.8 ± 1.2	
Model 1	0.96 (0.87–1.06)	1.0	0.83 (0.70–0.99)	0.91 (0.76–1.08)	0.83 (0.59–1.16)	0.39
Model 2	0.95 (0.86–1.06)	1.0	0.84 (0.59–1.20)	0.96 (0.67–1.37)	0.84 (0.58–1.20)	0.46
Model 3	0.97 (0.88–1.08)	1.0	0.89 (0.62–1.27)	1.01 (0.70–1.45)	0.88 (0.61–1.27)	0.64
**Percentage uncarboxylated osteocalcin**						
	**Per 5% increment**	**Q1**	**Q2**	**Q3**	**Q4**	**P**_**trend**_
%ucOC		27.3 ± 6.0	41.8 ± 3.3	52.4 ± 2.9	66.2 ± 7.0	
Model 1	1.00 (0.97–1.05)	1.0	0.68 (0.57–0.81)	0.72 (0.61–0.86)	1.05 (0.75–1.46)	0.80
Model 2	1.01 (0.97–1.06)	1.0	0.70 (0.49–0.99)	0.76 (0.54–1.09)	1.07 (0.75–1.54)	0.69
Model 3	1.01 (0.97–1.06)	1.0	0.71 (0.50–1.02)	0.78 (0.55–1.12)	1.09 (0.76–1.57)	0.64

Data are Odds Ratio’s (95%CI), Q = Quartile

Model 1: waist circumference, sex, age and cohort adjusted.

Model 2: additionally adjusted for smoking status, family history, hypertension, alcohol, physical activity and education.

Model 3: additionally adjusted for total energy intake, and energy adjusted intakes of saturated fat, mono- and poly unsaturated fat, fiber, protein and calcium

Because a borderline significant interaction term was observed between %ucOC and sex (p = 0.07), these analyses were stratified by sex. In women, a higher %ucOC tended to be associated with an increased risk of type 2 diabetes (P_trend_ = 0.08) with an OR_Q4_ of 1.36 (0.89–2.09) in the multivariable adjusted model. In continuous analyses, each 5% increment of %ucOC was associated with an increased risk of diabetes with an OR of 1.05 (1.00–1.11). In men, no association between the %ucOC and the risk of type 2 diabetes was found.

Waist circumference was the strongest confounder in the association between osteocalcin and type 2 diabetes. When waist circumference was replaced by BMI, none of the osteocalcin forms were associated with risk of type 2 diabetes with an OR_Q4_ of 0.92 (0.64–1.32) for tOC, 0.84 (0.59–1.21) for ucOC and 1.05 (0.73–1.50) for %ucOC in the final model. The interaction between sex and osteocalcin was no longer significant (p = 0.14).

The correlation between vitamin K intake and %ucOC was -0.05 (p = 0.04), with similar results for phylloquinone (r_s_ = -0.05) and menaquinones (r_s_ = -0.05). The level of %ucOC was not correlated with tOC levels (r_s_ = 0.02, p = 0.46) while the correlation between ucOC and tOC was 0.8 (p<0.001). The correlation between %ucOC and ucOC was 0.53 (p<0.001).

## Discussion

The purpose of this study was to investigate the associations between the different forms of osteocalcin and risk of type 2 diabetes. TOC, ucOC and %ucOC were not associated with the risk of type 2 diabetes. Furthermore, %ucOC was not correlated with vitamin K intake from the FFQ.

To date, only four prospective studies have investigated the association of different osteocalcin forms with diabetes incidence [4,11–13]. For tOC, three prospective studies investigated the association between tOC and the risk of type 2 diabetes [11–13]. A meta-analysis of these prospective cohort studies showed a pooled results of 0.89 (0.78–1.01) [10]. Only one small scale study showed that higher tOC concentrations were associated with lower diabetes incidence (OR 0.90 (0.81–0.99)) [12]. The reason for the discrepancy with our results is unclear. Our study is the first that included both men and women. When we stratified this association for sex, we could not detect an association between tOC and the risk of type 2 diabetes among both women and men as well. It is therefore unlikely that this explains any differences with previous studies. The most likely explanations are the differences in sample size and assay methods. This is the first study using the HAP binding assay to measure osteocalcin levels. The different assays may have resulted in different baseline osteocalcin levels. Furthermore, this is the first large-scale study, while previous studies consisted of small sample sizes with low event rates.

For absolute levels of ucOC, only one previous study of 359 men and women at high risk of cardiovascular disease showed that lower ucOC was associated with an increased risk of type 2 diabetes (OR 1.68 (1.09–2.59) T1 versus T3) [4]. We could not detect an association of ucOC with diabetes risk, which is comparable to the study of Ngarmukos et al [12]. The inconsistency may be due to differences in study subjects because the association was only observed in participants at high cardiovascular risk.

However, because of the high correlation between absolute levels of tOC with cOC and ucOC, different assays to measure osteocalcin cannot distinguish the differences between the different carboxylated forms. The study of Ngarmukos et al., of 63 cases and 63 controls explored the relationship between the ratio of ucOC/tOC, reflecting %ucOC, with the risk of type 2 diabetes [12]. However, this study was too small to estimate effects about ucOC/tOC ratio and the risk of type 2 diabetes [12]. This study also used two different assays to measure ucOC and tOC. Because these measurements are not standardized equivalently, it is not an adequate measurement to estimate %ucOC [5]. Only the cross sectional study by Yeap et al. used the HAP assay and showed a borderline significant association between ucOC/tOC ratio and type 2 diabetes risk (OR 1.11, p = 0.06) [14]. However, the study of Yeap et al. is cross-sectional and only included men. Since our study is the first investigating the association between %ucOC and diabetes risk in women, we are not able to compare the borderline significant association with previous studies.

Several explanations could play a role in the different observations among men and women in our study. Since these results were only apparent after adjustment for waist circumference, it could be driven by central adiposity. Waist circumference is a better measure of central obesity compared to BMI and is more strongly associated with diabetes incidence among women than men [24]. TOC was moderately associated with waist circumference (r_s_ = -0.36) and BMI (r_s_ = -0.23) in men, but not in women (r≈-0.09). However, %ucOC was not associated with waist circumference or BMI in both men and women (r≈-0.05), which makes it unlikely to explain our findings. Furthermore, our study consisted of only 22.9% men, giving less power to detect an association in men than women. However, when waist circumference was replaced by BMI, the borderline significant association between %ucOC and diabetes incidence in women disappeared and no association between any of the osteocalcin forms and diabetes incidence was present in both men and women. This makes it likely that the borderline significant association for women could simply be a chance finding.

Previous studies suggested %ucOC as a marker of vitamin K intake, but we could not confirm these findings. The correlation between %ucOC and vitamin K intake in our study was much lower than in previous studies, which showed correlations of -0.21 and -0.33 respectively [25,26]. However, in these studies vitamin K intake was measured with 3 day food- records before the day of blood examination [25,26]. The validity of the FFQ may explain the difference between our study and previous studies. The relative validity of menaquinones based on the FFQ was reasonable (r = 0.60 and 0.48 in men and women), but for phylloquinone the validity was relatively low (r = 0.26 and 0.19 in men and women respectively). This may explain the low correlation between phylloquinone intake and osteocalcin forms, but not for menaquinones. Another explanation could be genetic variation in vitamin K metabolism. A previous study showed that the correlation between menaquinone-7 intake and ucOC and tOC ratio differed by polymorphisms of the gamma glutamyl carboxylase gene [26].

The strengths of this study include its prospective design, large sample size, verification of diabetes cases against medical records and the use of %ucOC. Most studies do not differentiate between the total and uncarboxylated forms of osteocalcin and use absolute levels. UcOC is highly correlated with tOC (r = 0.8). Since tOC is dependent of osteoblast activity, it is difficult to differentiate the osteoblastic effect of osteocalcin from the role of the vitamin K dependent carboxylation in these studies. For this reason, %ucOC is preferred as marker for vitamin K intake [5,27]. Besides the strengths, this study also has some limitations which need to be addressed. We were able to correct for many diabetes risk factors but as in many observational studies, residual confounding may be present. Second, osteocalcin levels and diabetes risk factors are only measured at baseline, therefore it was not possible to evaluate whether diabetes risk factors changed overtime.

In summary, this study suggests that TOC, ucOC and %ucOC are not associated with the risk of type 2 diabetes. However, more large scale cohort studies are needed to clarify the presence of any association between the different forms of osteocalcin and the risk of type 2 diabetes.

## Supporting Information

S1 File (DOCX)

## References

[1] Cranenburg ECM, Schurgers LJ, Vermeer C. Vitamin K: The coagulation vitamin that became omnipotent. Thromb Haemost. 2007;98: 120–125. 17598002

[2] Schurgers LJ, Teunissen KJF, Hamulyák K, Knapen MHJ, Vik H, Vermeer C. Vitamin K-containing dietary supplements: Comparison of synthetic vitamin K1 and natto-derived menaquinone-7. Blood. 2007;109: 3279–3283. 17158229 10.1182/blood-2006-08-040709

[3] Vermeer C. Vitamin K: The effect on health beyond coagulation—An overview. Food and Nutrition Research. 2012. 10.3402/fnr.v56i0.5329 PMC3321262 22489224

[4] Díaz-López A, Bulló M, Juanola-Falgarona M, Martínez-González MA, Estruch R, Covas M-II, et al Reduced serum concentrations of carboxylated and undercarboxylated osteocalcin are associated with risk of developing type 2 diabetes mellitus in a high cardiovascular risk population: a nested case-control study. J Clin Endocrinol Metab. 2013;98: 4524–4531. 10.1210/jc.2013-2472 24037881

[5] Gundberg CM, Lian JB, Booth SL. Vitamin K-Dependent Carboxylation of Osteocalcin: Friend or Foe? Adv Nutr An Int Rev J. 2012;3: 149–157. 10.3945/an.112.001834 PMC3648715 22516722

[6] Bullo M, Moreno-Navarrete JM, Fernandez-Real JM, Salas-Salvado J. Total and undercarboxylated osteocalcin predict changes in insulin sensitivity and beta cell function in elderly men at high cardiovascular risk. Am J Clin Nutr. 2012;95: 249–255. 10.3945/ajcn.111.016642 22170359

[7] Lee NK, Sowa H, Hinoi E, Ferron M, Ahn JD, Confavreux C, et al Endocrine Regulation of Energy Metabolism by the Skeleton. Cell. 2007;130: 456–469. 17693256 10.1016/j.cell.2007.05.047 PMC2013746

[8] Shea MK, Gundberg CM, Meigs JB, Dallal GE, Saltzman E, Yoshida M, et al Gamma-carboxylation of osteocalcin and insulin resistance in older men and women. Am J Clin Nutr. 2009;90: 1230–1235. 10.3945/ajcn.2009.28151 19776145 PMC2762158

[9] Choi HJ, Yu J, Choi H, An JH, Kim SW, Park KS et al Vitamin K2 supplementation improves insulin sensitivity via osteocalcin metabolism: a placebo-controlled trial. Diabetes Care. 2011;34: e147–e. 10.2337/dc11-0551 21868771 PMC3161300

[10] Kunutsor SK, Apekey TA, Laukkanen JA. Association of serum total osteocalcin with type 2 diabetes and intermediate metabolic phenotypes: systematic review and meta-analysis of observational evidence. Eur J Epidemiol. 2015; 10.1007/s10654-015-0058-x 26085114

[11] Liatis S, Sfikakis PP, Tsiakou A, Stathi C, Terpos E, Katsilambros N, et al Baseline osteocalcin levels and incident diabetes in a 3-year prospective study of high-risk individuals. Diabetes Metab. 2014;40: 198–203. 10.1016/j.diabet.2014.01.001 24529960

[12] Ngarmukos C, Chailurkit LO, Chanprasertyothin S, Hengprasith B, Sritara P, Ongphiphadhanakul B. A reduced serum level of total osteocalcin in men predicts the development of diabetes in a long-term follow-up cohort. Clin Endocrinol (Oxf). 2012;77: 42–46. 10.1111/j.1365-2265.2011.04215.x 21916911

[13] Hwang YC, Ahn KJ, Jee JH, Chung HY, Jeong IK, Lee MK. Circulating osteocalcin level is not associated with incident type 2 diabetes in middle-aged male subjects: Mean 8.4-year retrospective follow-up study. Diabetes Care. 2012;35: 1919–1924. 10.2337/dc11-2471 22773701 PMC3424992

[14] Yeap BB, Alfonso H, Paul Chubb S a, Gauci R, Byrnes E, Beilby JP, et al Higher serum undercarboxylated osteocalcin and other bone turnover markers are associated with reduced diabetes risk and lower estradiol concentrations in older men. J Clin Endocrinol Metab. 2014; jc20143019. 10.1210/jc.2014-3019 25365314

[15] Beulens JWJ, Monninkhof EM, Verschuren WMM, van der Schouw YT, Smit J, Ocke MC, et al Cohort profile: the EPIC-NL study. Int J Epidemiol. 2010;39: 1170–8. 10.1093/ije/dyp217 19483199

[16] Sluijs I, van der A DL, Beulens JWJ, Spijkerman AMW, Ros MM, Grobbee DE, et al Ascertainment and verification of diabetes in the EPIC-NL study. Neth J Med. 2010;68: 333–339. 20739736

[17] Wareham NJ, Jakes RW, Rennie KL, Schuit J, Mitchell J, Hennings S, et al Validity and repeatability of a simple index derived from the short physical activity questionnaire used in the European Prospective Investigation into Cancer and Nutrition (EPIC) study. Public Health Nutr. 2003;6: 407–413. 12795830 10.1079/PHN2002439

[18] Schurgers LJ, Vermeer C. Determination of Phylloquinone and Menaquinones in Food Effect. Pathophysiol Haemost Thromb. 2001;30: 298–307. 10.1159/000054147 11356998

[19] Shearer MJ, Bach A, Kohlmeier M. Chemistry, nutritional sources, tissue distribution and metabolism of vitamin K with special reference to bone health. J Nutr. 1996;126: 1181S–6S. Available: http://www.ncbi.nlm.nih.gov/pubmed/8642453 8642453 10.1093/jn/126.suppl_4.1181S

[20] Booth SL. Roles for vitamin K beyond coagulation. Annu Rev Nutr. 2009;29: 89–110. 10.1146/annurev-nutr-080508-141217 19400704

[21] Willett WC, Howe GR, Kushi LH. Adjustment for total energy intake in epidemiologic studies. Am J Clin Nutr. 1997;65: 1220S–1228S; discussion 1229S–1231S. 9094926 10.1093/ajcn/65.4.1220S

[22] Rubin DB. Multiple Imputation after 18+ Years. J Am Stat Assoc. Taylor & Francis; 1996;91: 473–489. Available: http://www.tandfonline.com/doi/abs/10.1080/01621459.1996.10476908

[23] Fawzy T, Muttappallymyalil J, Sreedharan J, Ahmed A, Alshamsi SOS, Al Ali MSSHBB, et al Association between Body Mass Index and Bone Mineral Density in Patients Referred for Dual-Energy X-Ray Absorptiometry Scan in Ajman, UAE. J Osteoporos. 2011;2011: 876309 10.4061/2011/876309 21772978 PMC3135277

[24] Langenberg C, Sharp SJ, Schulze MB, Rolandsson O, Overvad K, Forouhi NG, et al Long-term risk of incident type 2 diabetes and measures of overall and regional obesity: the EPIC-InterAct case-cohort study. PLoS Med. 2012;9: e1001230 10.1371/journal.pmed.1001230 22679397 PMC3367997

[25] Haraikawa M, Tsugawa N, Sogabe N, Tanabe R. Effects of gamma-glutamyl carboxylase gene polymorphism (R325Q) on the association between dietary vitamin K intake and gamma-carboxylation of osteocalcin in young adults. 2013;22: 646–654. 10.6133/apjcn.2013.22.4.01 24231026

[26] Sogabe N, Tsugawa N, Maruyama R, Kamao M, Kinoshita H, Okano T, et al Nutritional effects of gamma-glutamyl carboxylase gene polymorphism on the correlation between the vitamin K status and gamma-carboxylation of osteocalcin in young males. J Nutr Sci Vitaminol (Tokyo). 2007;53: 419–425. 18079608 10.3177/jnsv.53.419

[27] Obrant KJ, Käkönen SM, Astermark J, Lilja H, Lövgren T, Akesson K, et al The proportion of carboxylated to total or intact osteocalcin in serum discriminates warfarin-treated patients from control subjects. J Bone Miner Res. 1999;14: 555–560. 10234576 10.1359/jbmr.1999.14.4.555

