# Supplementary material for: Osteocalcin Is Not Associated with the Risk of Type 2 Diabetes: Findings from the EPIC-NL Study
Source: PLoS One. 2015 Sep 29;10(9):e0138693. doi: 10.1371/journal.pone.0138693 (PMC4587948; doi:10.1371/journal.pone.0138693)
Supplement: S1 File — (DOCX) [file pone.0138693.s001.docx]

**Supporting information**

The hydroxyl apatite (HAP) method is based on lower affinity of ucOC for hydroxyapatite compared to the cOC. To determine the relative amount of ucOC in an individual serum sample, 100 µL unknown serum is added to 100 µL of a 3 mg/mL suspension of hydroxyapatite in Tris buffer. The tubes are shaken vigorously for 1 h at room temperature and then centrifuged for 2 min in a microfuge (10,000 x *g*). The supernatant is removed, and the amount of unbound osteocalcin measured by RIA as described below.

TOC and ucOC are then measured by a standard equilibrium radioimmunoassay. Assay buffer consists of 0.01 M NaH_2_PO_4_, 0.125 M NaCl, 0.1% Tween, 0.025 M Na_4_EDTA, and 0.1% bovine serum albumin, pH 7.4. All assays consist of specific antiserum 1:10,000 final dilution, carrier non-immune serum, 25 µL of a known concentration of standards ranging from 1 ng/ml to 75 ng/ml in triplicate, unknown serum samples, hydroxyapatite supernatant and quality control samples in duplicate, and 2 x 10^4^ cpm of ^125^I- osteocalcin in a final volume of 0.50 ml. The mixture is incubated for 18-24 hours at room temperature and terminated by precipitation with donkey anti-rabbit IgG antiserum (Pelfreeze, Rogers AR). Bound fractions are counted in a Beckman automated gamma counter linked to a computer. The B/B_o_ ratio of each standard and sample is corrected for non-specific binding. Assay characteristics are computer derived for each assay. Inter- and intra-assay coefficients of variation are 6.7 % and 3.2% respectively. %ucOC is calculated from measured tOC and ucOC.
